# Annual Address before the Ohio State Dental Society

**Published:** 1869-12

**Authors:** W. P. Horton

**Affiliations:** President


### DENTAL REGISTER.

VOL. XXIII.] DECEMBER, 1869. [No. 12

### Original Communications.

---

### ANNUAL ADDRESS BEFORE THE OHIO STATE DENTAL SOCIETY.

BY THE PRESIDENT, DR. W. P. HORTON.

*Gentlemen of the Ohio State Dental Society:*

In again resuming business it is eminently proper that I make a few suggestions as to the needs of the profession in this State, I might say the profession at large, and which it may be in the power of this body to supply, or if not fully supply, at least put the profession in the way of obtaining for itself. I need not trench upon the rights or duties of the Corresponding Secretary or Executive Committee. They have well done their part in arranging the order of business and attending to our comfort and convenience during our sittings. These are very essential to facilitate our deliberations and enable us properly to consider and digest the work before us.

Neither do I wish in my official capacity to set up my dictum against the better or deliberate judgment of this body in any way. But only to call your attention to matters which need your earnest consideration aside from the regular order of business, and by the aid of special or standing committees, to arrive at such conclusions as will tend to the enlightment of the profession at large; the onward and upward growth of ourselves; and the record of which in future years we shall not be ashamed to look back upon, or have our successors look upon and call our own. The first subject to which I will call your attention is "The law regulating the practice of Dentistry in this State." The law was designed to promote the general good of Dentist and patient, and I am certain is a good one so far as it goes.

Perhaps it *goes* far enough to secure all needed rights to the profession and protection to the people, were it executed. But I am as certain that an influence eminating from those nominally in the profession is weighing down the law to such an extent that very few of their patients know that any law exists, much less one that will in any way afford them present or future protection. This silent opposition comes from men, most of whom never spent much time in studying that which they attempt to practice, and pretend to think it useless for others to be compelled to do what they were *not*, no matter who suffers at their hands, and the hands of ignorance in general, so long as they, the sufferers, pay their money freely, and do not complain too loudly of the kind and quality of work they receive in return. These practitioners (shall I call them) go upon the general principle that "when ignorance is bliss it is folly to be wise," either for operator or operatee. Without any attempt at argument in the premises I merely call your attention to the fact that I have learned of no prosecution under the law, while I have been informed that many are practicing in violation of its precepts.

The next subject that in my judgment needs your attention is "The Code of Ethics." Right at this point and upon this subject, I wish no misunderstanding, and will therefore say that it is not my intention to make a raid upon our code of ethics. On the contrary, I am personally in favor of a code in general, and this one in particular.

That a code of ethics is needed for the government of the members of our profession in their intercourse with each other, professionally, there seems to be no doubt in the minds of a large and respectable portion of its members. On the other hand, there are very many who, while in our meetings, say nothing against it, and thereby tacitly acquiesce in its provisions, when in their offices and in their daily walk and conversation, either silently ignore, loudly inveigh against, or openly and boldly put it at defiance.

There is another class who rarely or never enter our social or scientific meetings, and who, in the communities where they reside, are reckoned as members of our profession. This class last though not least, numbering fully one-half of those called Dentists, can not be easily reached either by human or divine laws. Many of these consider themselves so well born that they totally ignore the necessity of regeneration, either in a temporal or spiritual point of view, and believing themselves wise beyond what is written, make themselves fools, not, however, beyond the hope of salvation. All laws to be of much practical importance, must derive their essential and germinal principle from a consent of the governed, or at least a respectable majority of the governed. Unless that be the case, the law, however, just it may be, either is trodden under foot as a dead letter, or becomes an element of interminable discord and confusion.

Would it not be well, therefore, to devise some means by which this contumacious class may be reached? and can it not be done by such a revision of both the laws referred to, as that a large majority of those nominally in the profession may cheerfully partake of their full benefits? In doing this I would in no wise lower our standard an iota. On the contrary let its motto ever be "Excelsior."

The third topic to which I wish to direct your attention is the struggle that has been and is still going on between the profession on one side, and an over shadowing and, in some particulars, unscrupulous monopoly on the other. I need not name the "Goodyear Dental Vulcanite Company," as most present have had too recent, and in many instances, too unpleasant reminders from the party to whom I refer to be in any doubt.

In what it is my purpose to present under this head, I shall not travel into the province of the committee appointed some three years ago, by a mass meeting of the members of this society and others, and charged with the duty of defending such members of the profession as complied with certain fixed conditions, and in accordance with the advice of its attorney, Col. S. S. Fisher, against the demands of this powerful monopoly. That committee will, without doubt, make a full and complete exhibit of what it has done, for your approval or rejection, as you may determine on final hearing. But the points I wish in particular to present are: First, Admitting that there can be at the present time no successful legal opposition to the claims of the Goodyear Company, the patents upon which these claims are based, expire in 1872, no very distant period in the future. Now, in carrying on a contest with your adversary there are at least two modes of warfare, which may be used separately or together, and either of which will give you almost incalculable advantage in its results viz: cut off his supplies, and then meet him in the field more than half way.

The first may be done by every Dentist in the nation, pertinaciously refusing to take a license from, or in any way pay tribute to this monster that stalks abroad like the very spirit of all evil, seeking a victim to appease the cravings of its rapacious maw. Whether this plan be feasible, or if it were whether it be advisable? are questions which I leave for you to decide. That it would be effectual in reducing the value of its stock to very low figures, in a very short space of time, there is little room for doubt.

The next move will be to prevent the renewal or any extension of the "Goodyear Patents," as far as their application to Dental purposes are concerned. I have already stated that the patents upon which the claims of this company rest as far as the Goodyear is concerned, expire in 1872. There are two of these, one covering the process and the other the product. These should never be renewed or extended, and a tax of five dollars on each Dentist in the nation would afford ample means to prevent any such consummation. But if a successful cruisade be carried on to that end there is no time to be lost. We must be first in the field, and with our armor brightly polished and fully adjusted, be prepared to "charge with all our chivalry."

Secondly, The Dental Vulcanite Company, after the expiration of the Goodyear patents, have to base its claims—in case the "Goodyear" be not extended—on the Cummings patent alone. This patent has been pronounced by competent lawyers to be possessed of no validity whatever. But as an effect against that it has the prestige in the "Weatherbee case," so called, of the opinion of one, at least, of the Judges of the Supreme Court of the United States. How far the hearing in that case was "exparte," I am not prepared to say. But that there were very important omissions in the testimony, especially on the part of the defense, there is no doubt in my mind, and I am of the opinion that most of the testimony which has arisen to the surface since the agitation of this matter has become so general, was not at the time of the hearing of the Weatherbee case, known to or within the reach of the defense. This testimony the profession can avail itself of in case coersion be resorted to on the part of the enemy. That legal coersion will be resorted to is as certain as that the present managers live, until in their opinion the proper time has come. Much reliance for success will be placed upon the fact that every Dentist who takes a license from the "Goodyear Dental Vulcanite Company" must subscribe to the validity of the Cummings as well as the Goodyear patent, or be debarred the privileges of either. How far the fact that these contracts were signed under "duress" will influence the Courts in favor of the defense, will be seen when the time of trial shall have fully come and the question passed upon. But there is no principle of law more clearly established than that such a contract is null and void. That being the case then the fight will be on the merits of the question of the validity of the Cummings patent per see.

Now I would respectfully suggest that the Dentists of this State and the United States combine, at least one year before the expiration of the "Goodyear" patent, and have their forces well armed, equipped and drilled, and at the first intimation of danger, advance their whole line upon the enemies works.

In making these suggestions I would not advocate over haste or illy advised opposition. But that the profession see eye to eye, and with the utmost cordiality and fraternal feeling, each bearing his own burthens at the proper time with cheerfulness, and with shoulder to shoulder, in one solid phalanx march on "from conquering unto conquer," until the very spirit of patent-rightism as applicable to Dental purposes be like Noah's dove, unable to find a resting place for the sole of its foot upon the face of this whole earth.

